# Disulfide bond-mediated stabilization of the oligomers of UDP-glucuronosyltransferase 2B7

**DOI:** 10.1016/j.jbc.2025.110502

**Published:** 2025-07-21

**Authors:** Yuu Miyauchi, Haruna Koba, Madoka Sawai, Hisao Kansui, Shinji Takechi, Eiji Hishinuma, Naomi Matsukawa, Masahiro Hiratsuka, Peter I. Mackenzie, Yuji Ishii

**Affiliations:** 1 Laboratory of Hygienic Chemistry, Faculty of Pharmaceutical Sciences, Sojo University, Kumamoto, Japan; 2 International University of Health and Welfare, School of Pharmacy at Fukuoka, Fukuoka, Japan; 3 Laboratory of Organic Chemistry, Faculty of Pharmaceutical Sciences, Sojo University, Kumamoto, Japan; 4 Tohoku Medical Megabank Organization, Tohoku University, Sendai, Japan; 5 Advanced Research Center for Innovations in Next-Generation Medicine, Tohoku University, Sendai, Japan; 6 Laboratory of Pharmacogenomics, Graduate School of Pharmaceutical Sciences, Tohoku University, Sendai, Japan; 7 Clinical Pharmacology, College of Medicine and Public Health, Flinders Medical Centre and Flinders University, Adelaide, Australia; 8 Division of Pharmaceutical Cell Biology, Graduate School of Pharmaceutical Sciences, Kyushu University, Fukuoka, Japan

**Keywords:** cysteine residue, disulfide, drug metabolism, gel electrophoresis, glucuronidation, intercellular cross-linking, mutagenesis, oligomerization, sodium dodecyl sulfate (SDS), Uridine 5′-diphospho-glucuronosyltransferase (UDP-glucuronosyltransferase)

## Abstract

UDP-glucuronosyltransferase (UGT) is an important drug-metabolizing enzyme involved in the detoxification of hydrophobic chemicals by conjugating them with hydrophilic glucuronic acid. Previous studies have revealed that UGTs form oligomers between or among the same or other isoforms, but the molecular mechanism underlying this formation remains unclear. In this study, we used optimized electrophoretic techniques to analyze UGT2B7 homo-oligomer formation. UGT2B7 was expressed in COS-1 cells, and lysates were analyzed by sodium dodecyl sulfate-polyacrylamide gel electrophoresis (SDS-PAGE). By omitting the heating and reducing steps during SDS-PAGE sample preparation, bands corresponding to dimer, tetramer, and higher-order oligomers were detected in addition to monomeric bands. Since these SDS-stable UGT2B7 oligomer bands disappeared with the addition of reducing agents, we hypothesized that intermolecular disulfide bonds are involved in the formation of UGT oligomers. The cysteine residues important for this oligomer formation were investigated. Analyses using alanine substitution and deletion mutants suggested that three cysteine residues of UGT2B7, Cys127, Cys156, and Cys282, are important not only for oligomer formation but also for glucuronidation ability. We further investigated the oligomerization of UGT2B7 in intact living cells using two membrane-permeable cross-linkers, disuccinimidyl suberate and dithiobis(succinimidyl propionate). UGT2B7-expressing cells were treated with these reagents and analyzed by western blot. This cross-linker treatment markedly reduced the UGT2B7 monomer band and increased the formation of higher-molecular-mass species. These results indicated that the majority of UGT2B7 is present within cells as oligomers, maintaining its enzymatic function, rather than as a monomer.

UDP-glucuronosyltransferase (UGT) plays a crucial role in drug metabolism, converting hydrophobic xenobiotics and endogenous substances to glucuronides (1). Approximately 20% of all drugs undergoing metabolism are conjugated by UGT. This contribution is the greatest of the phase II drug-metabolizing enzymes and is second only to that of cytochrome P450 (P450) (2). Despite this large contribution to drug metabolism, UGT structure research has lagged far behind that for P450. One reason for this is the difficulty in producing crystals of the full-length protein, since purifying UGT that retains enzyme activity has only been demonstrated for recombinant human UGT1A9 with a hexa-histidine-tag (3). Despite this, the partial structure of the substrate-binding region of UGT2B7 has been determined using nuclear magnetic resonance (NMR) (4), and its UDP-glucuronic acid (UDPGA)-binding domain has been determined using crystallography (5) with the maltose-binding protein as a tag. There is no established method for the purification of recombinant UGT without an epitope tag, and it is not as easy to purify it from animal tissues as it is to purify other drug-metabolizing enzymes. Hence, in the case of UGT, the crystal-structure-based approach that was effective for P450 cannot be applied for full-length UGT, and classical approaches must be used for analyses of its enzymatic and functional properties.

UGT is a type-I membrane protein located in the endoplasmic reticulum (ER) as shown in Figure 1*A*. It is predicted that most of the protein is oriented toward the lumen with a transmembrane region and a cytoplasmic tail consisting of approximately 20 amino acids at the C-terminus (6). One major characteristic of UGTs is that they form oligomers between the same and different isoforms, as first noted when purifying UGT from animal tissues in early studies. For example, Tukey and Tephly suggested that two UGT isoforms, which catalyze the glucuronidation of estrone or *p*-nitrophenol, form a tetramer because when they tried to purify these UGTs from rabbit liver, the final products had an M_r_ of 230,000 in gel chromatography, which is four times larger than that predicted size of the monomer (M_r_ 57,000) (7). Strong support for UGT oligomer formation was provided in more recent studies using several different methods. Ikushiro *et al.* used isoform-specific antibodies and a cross-linker, 1,6-bis(maleimido)hexane, to show that UGT1A isoforms form heterodimers with UGT2B1 in rat liver microsomes (8). Hetero-oligomers of UGTs (UGT1A1, UGT1A6, and UGT2B7) were similarly confirmed in human liver microsomes by co-immunoprecipitation with isoform-specific antibodies (9). In addition, radiation inactivation (10), yeast two-hybrid assays (11), sodium dodecyl sulfate-polyacrylamide gel electrophoresis (SDS-PAGE) (12, 13, 14), and fluorescence resonance energy transfer (FRET) have also been applied to demonstrate UGT oligomerization (15). Notably, the FRET results indicated that UGT oligomerization can occur in the intact ER of living cells rather than during the sampling process of microsomes or homogenates.

**Figure 1:**
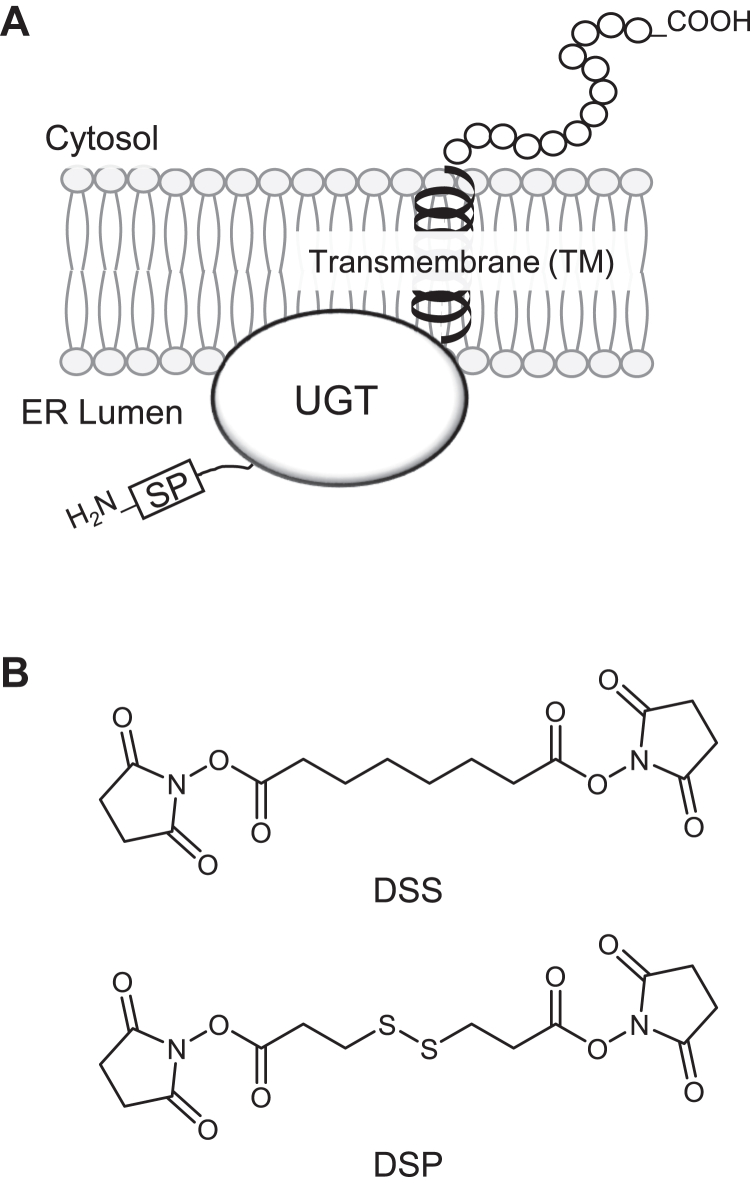
**Structures of UGT and Cross-linkers.***A*, postulated structure of UGT in the ER membrane. SP, signal peptide. *B*, cross-linkers utilized in this study. DSS, disuccinimidyl suberate; DSP, dithiobis(succinimidyl propionate).

Although many studies have supported the formation of UGT oligomers, as described, the detailed molecular mechanisms underlying the oligomerization remain unclear. To address this issue, in this study, we selected SDS-PAGE among the reported methods and optimized the experimental conditions for UGT oligomer analysis. Further, two chemical cross-linkers (Fig. 1*B*), disuccinimidyl suberate (DSS) and dithiobis(succinimidyl propionate) (DSP), were applied to enable analysis by SDS-PAGE while maintaining the oligomeric state of UGT2B7 in living cells.

## Results

### UGT2B7 oligomers were detected using non-reducing SDS-PAGE

**Figure 2:**
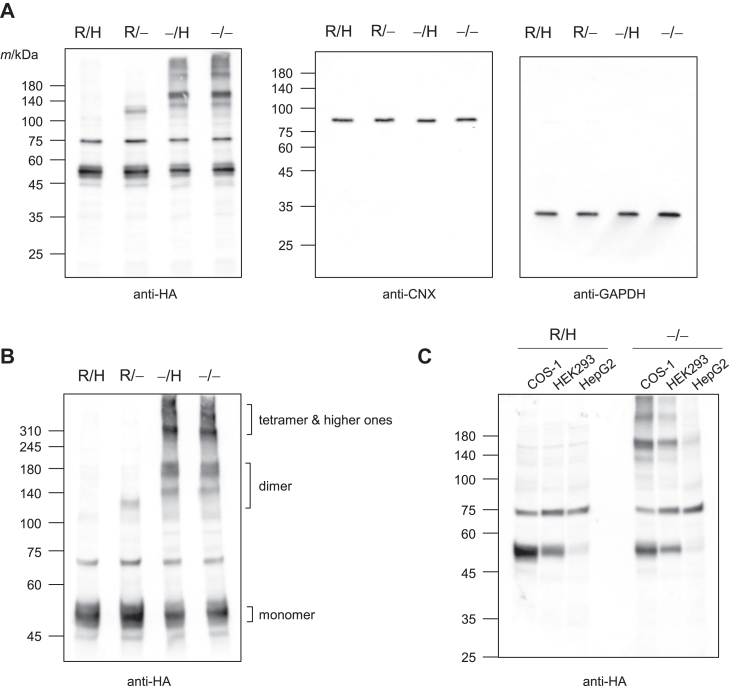
**Determination of PAGE conditions to detect UGT2B7 homo-oligomers by** w**estern blotting.***A*, cell lysates expressing UGT2B7-HA (50 μg) were separated by SDS-PAGE for 100 min in the presence and absence (−) of either the reducing agent (R) or the heating step (H). UGT2B7-HA was detected with an anti-HA antibody, and endogenous CNX and GAPDH were also detected as controls. *B*, separation of bands of high molecular weight by electrophoresis for a long time (150 min). *C*, UGT2B7-HA was expressed in HEK293 and HepG2 cells, as same as COS-1 cells, and prepared lysates (50 μg) were separated by SDS-PAGE performed under conditions where reducing agent and a heating step were used (R/H) or omitted (−/−).

In SDS-PAGE, sample proteins are usually denatured to linear peptides by SDS, a reducing reagent, and heating (16). COS-1 cell lysates expressing UGT2B7 fused with a C-terminal hemagglutinin tag (UGT2B7-HA, HA-tag; YPYDVPDYA) were subjected to electrophoresis in the presence and/or absence of reducing agent and heating (four conditions) before the detection of UGT2B7-HA by western blotting. When heating was omitted, only a few bands corresponding to dimers (M_r_ 100,000–120,000) were detected, but when the reducing agent was omitted, the change was marked, and bands corresponding to postulated dimers, tetramers, and higher-order oligomers were detected (Fig. 2*A*, anti-HA). In contrast, there was no difference in the detection of either calnexin (CNX), an ER protein that has the same membrane topology as UGT, or glyceraldehyde 3-phosphate dehydrogenase (GAPDH) under the four conditions, with only a single band observed for each (Fig. 2*A*; anti-CNX and anti-GAPDH). In SDS-PAGE, SDS disrupts the conformation of the protein, and oligomers are generally dissociated and detected as monomers. GAPDH was detected only as a monomer under the four conditions in which SDS was present, although GAPDH is also known to form oligomers, and the tetramer is especially important for glycolytic function (17, 18). Thus, these results suggested that UGT2B7 forms unique oligomers that are stable to SDS and that a disulfide bond(s) contributes to the stabilization of the oligomers. We extended the electrophoresis time to better separate UGT2B7 oligomers (Fig. 2*B*). These results suggested that the band corresponding to the UGT2B7 dimer has multiple forms with molecular weights ranging from 120,000 to 200,000. The tetramers and higher-order oligomers were estimated to have molecular weights of 300,000 or more, and further separation would be difficult to achieve by simply extending the electrophoresis time. We further confirmed that SDS-stable UGT oligomer formation was observed not only in COS-1 cells but also in HEK293 and HepG2 cells, which are commonly used to study drug-metabolizing enzymes (Fig. 2*C*).

### Cys127 and Cys156 are important for the formation of disulfide bonds between UGT2B7 monomers

**Figure 3:**
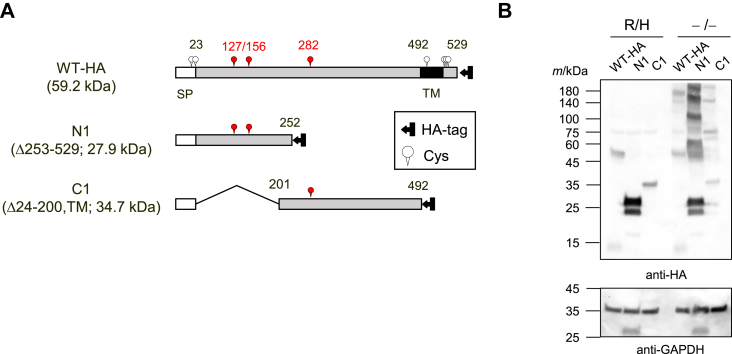
**Identifying the cysteine residue(s) involved in intermolecular disulfide bond formation in the luminal domain of UGT2B7.***A*, schematic sequence of UGT2B7 wild-type-HA (WT-HA) and the two deletion mutants, N1 (Δ253–529) and C1 (Δ24–200, TM), in which cysteine residues are indicated. Cys127, Cys156, and Cys282 are highlighted in *red*. The molecular mass of each monomer is predicted and shown. *B*, analysis of oligomers of WT-HA, N1, and C1 by western blotting. Cell lysates expressing each target (50 μg) were separated by SDS-PAGE performed under conditions where reducing agent and a heating step were used (R/H) or omitted (−/−). WT-HA and mutant UGT2B7 were detected using an anti-HA antibody, and GAPDH was detected as a control. SP, signal peptide; TM, transmembrane region.

**Figure 4:**
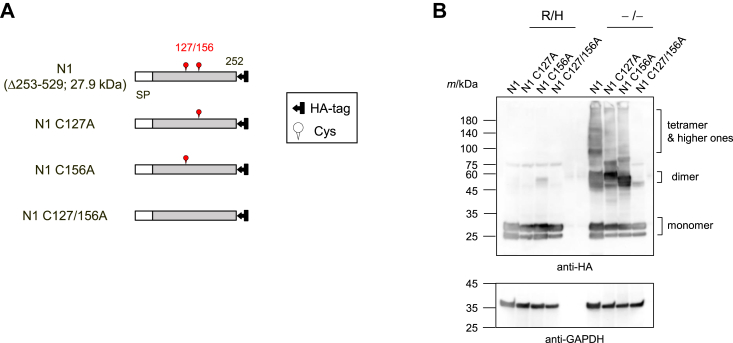
**Effect of alanine-substitutions of Cys127 and Cys156 on oligomer formation.***A*, schematic sequences of the N1 mutant and its alanine-substituted mutants in which both or either Cys127 or Cys156 were mutated. The molecular mass of N1 mutant is predicted and shown. SP, signal peptide. *B*, analysis of oligomers of N1, N1 C127A, N1 C156A, and N1 C127/156A by western blotting. Cell lysates expressing each target (50 μg) were separated by SDS-PAGE performed under conditions where a reducing agent and a heating step were used (R/H) or omitted (−/−). N1 and the N1-based mutants were detected using an anti-HA antibody, and GAPDH was detected as a control.

To determine the cysteine residues that form inter-subunit disulfide bonds, mutants of UGT2B7 were systematically designed and analyzed for oligomer formation using SDS-PAGE in the absence of the heating step and reducing agent. The UGT2B7 protein contains nine cysteine residues (Fig. 3*A*; WT, wild-type), of which Cys17 and Cys23 are present in the signal peptide (1–23 region) and are thus eventually removed. In addition, several previous studies have indicated that the lack of the transmembrane domain (493–510 region) and cytoplasmic tail (511–529 region) does not affect UGT oligomer formation (19, 20, 21, 22); thus, it is unlikely that the four cysteine residues, Cys500, Cys511, Cys512, and Cys515, located in these domains are essential for disulfide bond formation. Hence, we focused on the three remaining cysteine residues, Cys127, Cys156, and Cys282, located in the luminal domain (24–492 region). An N1 mutant (Δ253–529) containing Cys127 and Cys156, and a C1 mutant (Δ24–200, TM) containing residue 282 were generated, expressed (Fig. 3*A*), and analyzed for the presence of SDS-stable oligomers (Fig. 3*B*). Bands corresponding to the predicted molecular weights of the respective monomers were observed in the presence of reducing agent and heating for both the WT and the two mutant proteins. In contrast, under the non-reducing and non-heating conditions, SDS-stable oligomers were detected for both mutants as well as the WT protein, suggesting that UGT2B7 has several oligomerization sites and that SDS-stable oligomers are formed depending on the three cysteines. To examine the importance of Cys127 and Cys156, we next analyzed alanine-substituted mutants, as shown in Figure 4*A*. N1 C127A, N1 C156A, and N1 C127/156A mutants are N1-based mutants in which the corresponding cysteine residues were replaced with alanine, either individually or simultaneously. The N1 mutant formed SDS-stable oligomers under non-reducing and non-heating conditions, but the formation of tetramers and higher-order oligomers was markedly reduced in the N1 C127A and/or N1 C156A proteins (Fig. 4*B*). Dimer formation was also altered in a mutation-dependent fashion for the single cysteine mutants, but was markedly decreased for the N1 C127/156A mutant. These results indicated that the presence of at least one of Cys127 or Cys156 in the region of amino acids 24 to 252 greatly stabilizes the UGT2B7 oligomer, especially the dimer, *via* disulfide bond formation. The bands at approximately 25 kDa corresponding to the N1 monomers were strong doublets (Fig. 3*B*). Given the strong reactivity with the antibody and the fact that the bands corresponding to the monomers of the N1-based mutants shown in Figure 4*B* also showed a similar doublet, we speculate that the doublets are both N1 monomers, and that the mutagenesis has made these doublets susceptible to some partial modification or degradation. Alternatively, introducing the mutation may have caused variations in the interactions between the monomer and SDS, resulting in different forms of mobility in the gel.

### Cys282 is also important in the formation of inter-subunit disulfide bonds

**Figure 5:**
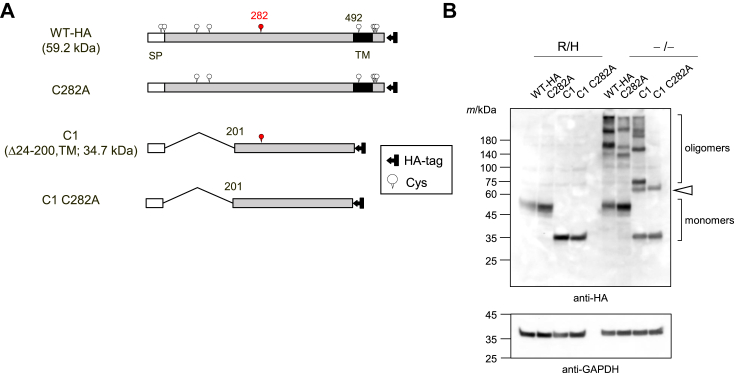
**Effect of alanine-substitution of Cys282 on oligomer formation.***A*, schematic sequence of wild-type-HA (WT-HA) and mutant UGT2B7, the C1 mutant, and their alanine-substituted mutants in which Cys282 was mutated. The molecular mass of WT-HA and C1 is predicted and shown. *B*, oligomers of WT-HA, C282A, C1, and C1 C282A proteins were analyzed by western blotting. Cell lysates expressing each target (50 μg) were separated by SDS-PAGE performed under conditions where a reducing agent and a heating step were used (R/H) or omitted (−/−). An anti-HA antibody was utilized to detect them. GAPDH was also detected as a control. The *white* arrow points to the band, which may be a system-dependent complex of the deletion mutant with another protein. SP, signal peptide; TM, transmembrane region.

**Figure 6:**
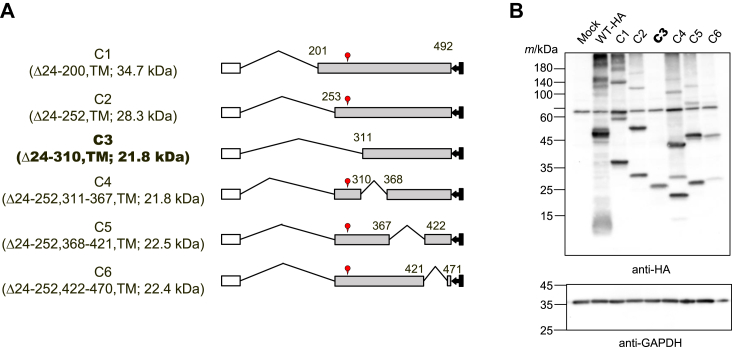
**Further investigation of the importance of Cys282 using serial deletion mutants.***A*, schematic sequence of the C1 mutants and the other deletion mutants (C2–C6). Among them, only the C3 mutant lacked Cys282. The molecular mass of each monomer is predicted and shown. *B*, cell lysates expressing each deletion mutant (50 μg) were separated by SDS-PAGE performed under conditions where a reducing agent and a heating step were omitted. The mutants were detected using an anti-HA antibody.

We next generated two mutants in which Cys282 was changed to alanine in both the WT and C1 mutant in order to estimate the importance of Cys282 in the formation of SDS-stable oligomers (Fig. 5*A*). In the presence of a reducing reagent and heating, no change was observed in the visibility of the bands corresponding to each monomer between WT and WT C282A or between C1 and C1 C282A proteins (Fig. 5*B*). In contrast, under non-reducing and non-heating conditions, alanine-substitution at Cys282 reduced SDS-stable oligomers of the WT protein and abolished them in the C1 mutant, which indicated that Cys282 is another important cysteine residue in the formation of intermolecular disulfide bonds in the 201 to 492 region of UGT2B7. Analysis of C1 and C1 C282A under non-heated and non-reduced conditions revealed a band at approximately 60 kDa, which was not observed under heated and reduced conditions (white arrowheads). This band was presumably due to the C1 mutant forming a complex with another protein in a Cys282-independent manner. A similar band was observed in Figure 3*B*, although the signal was weak. To support this result, deletion mutants of the 253 to 470 region, named C2–C6 (Fig. 6*A*), that were systematically generated in our previous study (23) were used. As all of these deletion mutants except C3 retain Cys282, we considered that these mutants would enable us to assess the importance of Cys282 in the formation of SDS-stable oligomers. As expected, all of the deletion mutants retained the ability to form SDS-stable oligomers (Fig. 6*B*), except C3, for which only the band corresponding to the monomer was observed, supporting the importance of Cys282 in the formation of inter-subunit disulfide bonds to stabilize UGT oligomers.

### Mutagenesis with full-length UGT2B7 supported the importance of the three cysteine residues

**Figure 7:**
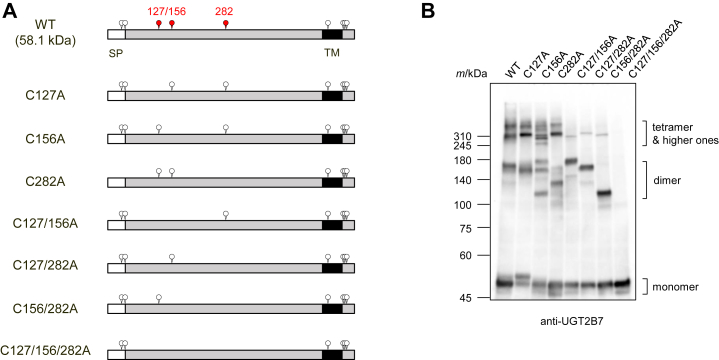
**Effect of alanine substitution of the three cysteine residues on oligomer stabilization using whole-length UGT2B7.***A*, schematic sequences of the systematic alanine-substituted mutants based on whole-length and HA-tag-free UGT2B7. The three cysteine target residues (Cys127, Cys156, and Cys282) are highlighted in *red* in UGT2B7 wild-type (WT). The molecular mass of WT is predicted and shown. *B*, cell lysates expressing each alanine-substituted mutant (50 μg) were separated by SDS-PAGE for 150 min and performed under conditions where a reducing agent and a heating step were omitted. The mutants were detected using an anti-UGT2B7 antibody. SP, signal peptide; TM, transmembrane region.

These results suggested that the three cysteine residues of UGT2B7 (Cys127, Cys156, and Cys282) are sites for disulfide bond formation in the SDS-stable oligomer. To investigate this, we used full-length and HA-tag-free UGT2B7 as a template, generated additional mutants in which the three cysteine residues were systematically replaced with alanine, and evaluated their ability to form oligomers (Fig. 7*A*). The effect on oligomers increased as the number of alanine substitutions increased (Fig. 7*B*). Simultaneous mutations at all three residues (C127/156/282A) greatly reduced oligomer formation of UGT2B7, and only monomeric bands were detected. While most of the tetrameric and higher bands were lost in the double mutants (C127/156A, C127/282A, and C156/282A), bands with molecular weights from 120,000 to 200,000, which increased compared to those in the WT, were detected. These are expected to be dimers formed by disulfide bonds between the remaining cysteine residue, for example. Cys127-Cys127 bound dimers in the C156/282A mutant, supporting the finding that dimers have several forms (Fig. 2*B*). Mutagenesis in a single cysteine residue resulted in stronger bands not seen in the WT, other than the dimer-derived bands seen with double mutagenesis, especially in C156A. The remaining two cysteine residues may form disulfide bonds in homo- and hetero-combinations, resulting in the formation of multiple oligomers. Cys127-Cys127, Cys127-Cys282, and Cys282-Cys282 bound dimers of C156 A. These results strongly support our hypothesis that these three cysteine residues are crucial for inter-subunit disulfide bonds.

### UGT2B7 exists mostly as oligomers in living cells

**Figure 8:**
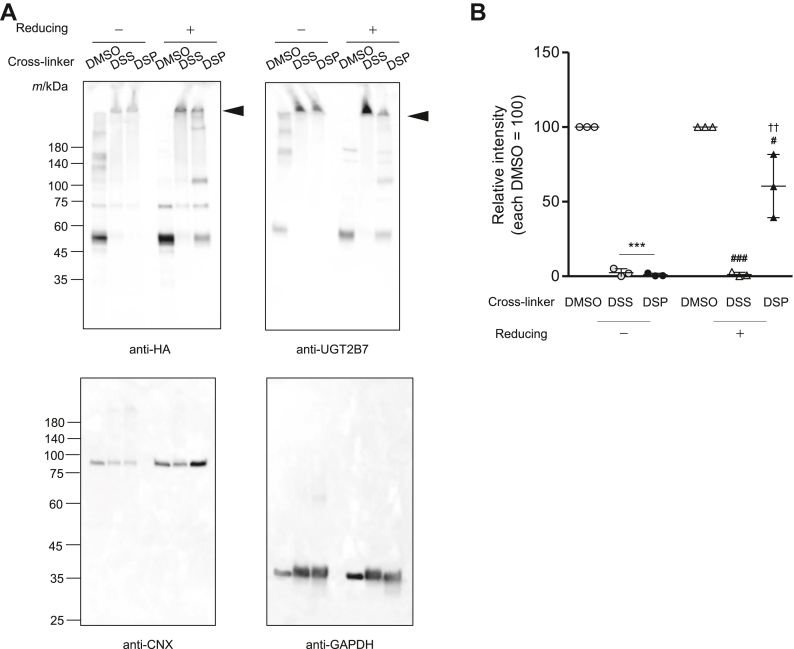
**Intracellular cross-linking of UGT2B7-HA with DSS and DSP.***A*, COS-1 cells transiently expressing UGT2B7-HA were treated with PBS containing 1 mM DSS/DSP for 30 min before lysate preparation. DMSO was used as a control. Cell lysates (50 μg) were separated by SDS-PAGE in the presence (+) or absence (−) of a reducing agent. UGT2B7-HA monomers and oligomers were detected using an anti-HA antibody and an anti-UGT2B7 antibody. As controls, endogenous CNX and GAPDH were detected. *Black arrows* indicate higher-order complexes of UGT2B7-HA formed in the cells. *B*, results of quantification of UGT2B7-HA monomer (n = 3). The blots of the repeated experiments are shown in Figure S1. The results of each quantification are illustrated in scatter plots, and the mean ± standard deviation of those values is shown. See the statistical analysis section of experimental procedures for details of the quantification. Statistical significance was determined by one-way analysis of variance, followed by Tukey’s test. ∗∗∗, *p* < 0.001 vs DMSO in the absence of reducing; ###, *p* < 0.001 and #, *p* < 0.05 vs DMSO in the presence of reducing, respectively; ††, *p* < 0.01 vs DSS in the presence of reducing.

As shown above, analyses of cell lysates indicated that UGT2B7 forms SDS-stable oligomers. Two cross-linkers were utilized to determine if this occurs *in vivo*. Both DSS and DSP are *N*-hydroxysuccinimide ester-based cross-linkers that are cell-membrane-permeable. When in the cell, they can form bridges between primary amino groups, including lysine residues, that are generally separated by 12 Å or less, thus stabilizing protein complexes that are already present in the cell without affecting native disulfide bounds. DSS forms non-cleavable cross-links, whereas DSP-mediated cross-links can be cleaved by reducing regents such as thiols (Fig. 1*B*). UGT2B7-HA was expressed in COS-1 cells, and the cells were treated with these cross-linkers before lysates were prepared. Samples were subjected to SDS-PAGE in the absence and presence of reducing agents before western blotting (Fig. 8*A*). Under non-reducing conditions, a band corresponding to the monomer was observed around M_r_ 55,000 in the dimethyl sulfoxide (DMSO)-treated cells, whereas this band was greatly reduced by DSS/SDP treatment, coincident with the appearance of higher-molecular-weight species (Fig. 8*A*, anti-HA left side). In contrast, under reducing conditions, bands corresponding to monomers reappeared in samples treated with DSP, which forms cleavable cross-links, whereas monomers were not recovered in samples treated with DSS (Fig. 8*A*, anti-HA right side). These changes were also confirmed by detection using an anti-UGT2B7 antibody (Fig. 8*A*, anti-UGT2B7). CNX and GAPDH were analyzed as controls; however, no such band migration upon DSS/DSP treatment was observed (Fig. 8*A*; anti-CNX and anti-GAPDH). The band intensities of the UGT2B7-HA monomer in the DMSO treatment were set as the baseline (100) to evaluate the effect of these cross-linking reagents (Figs. 8*B* and S1). In the absence of the reducing reagent, the amount of monomer was significantly reduced in the DSS/DSP treatments compared to that in the DMSO treatment, and almost none was observed. In contrast, in the presence of a reducing agent, there was a significant difference in the amount of UGT2B7-HA monomer between the DSS and DSP treatments, with the monomer recovering to approximately 70% of that in the DMSO treatment in the DSP treatment, compared to almost zero in the DSS treatment. These results indicated that most of the UGT2B7 protein exists as oligomers, rather than monomers, in living cells and that physical or chemical processes during sample preparation cause oligomers that are not stabilized by disulfide bonds to dissociate into monomers.

### The alanine substitution of any of the three cysteines abolished the glucuronidation abilities of UGT2B7

**Figure 9:**
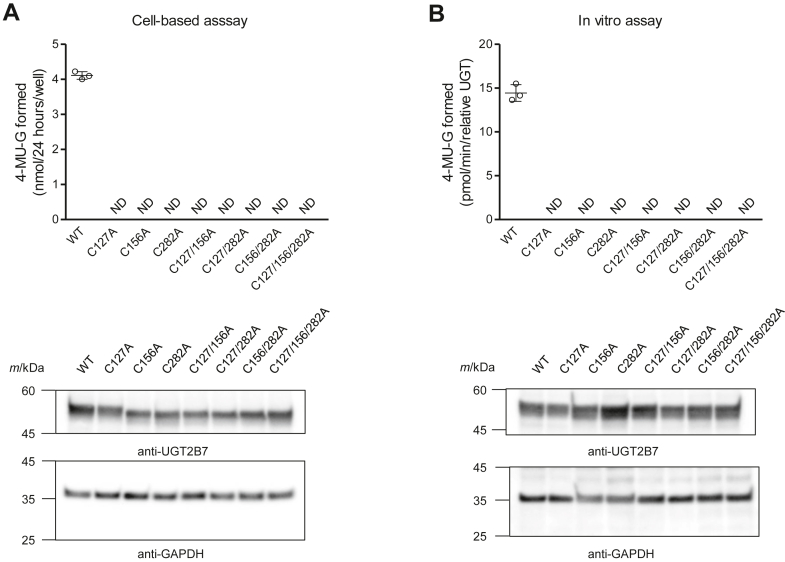
**Glucuronidation abilities of the alanine-substituted mutants.***A*, each alanine-substituted mutant was expressed in COS-1 cells and their glucuronidation abilities were measured using a cell-based approach. Briefly, the culture medium was replaced to DMEM containing 500 μM 4-MU 48 h after transfection. The cells were maintained for 24 h, and a portion of the culture medium (300 μl) was collected and mixed with 150 μl of methanol. Generated 4-MUG was analyzed with HPLC. The results of each triplicate assay are illustrated in scatter plots, and the mean ± standard deviation of those values are shown. The cells expressing UGT2B7 or its mutants were lysed, and the lysates (20 μg) were subjected to western blot to compare expression levels of the UGTs. *B*, the catalytic abilities of the mutants were measured using an *in vitro* approach. Cell homogenates were prepared from COS-1 cells expressing UGT2B7 or its mutants and used as an enzyme source. The homogenates (300 μg) were treated with a pore-forming peptide, alamethicin, mixed with 4-MU and UDPGA, and incubated for 1 hour at 37 °C. Enzyme reaction was stopped by adding methanol, and proteins were removed by centrifugation. The obtained supernatants were collected and analyzed with HPLC. See the UGT assay section of experimental procedures for details of the assay. The expression levels of UGT2B7 and its mutants were analyzed *via* western blots. ND, not detectable.

Because UGT is the enzyme responsible for the detoxifying drugs and toxic compounds, it is important to address whether oligomer formation affects its catalytic ability. We measured the glucuronidation activity of the generated alanine-substituted mutants (Fig. 7*A*) using two methods. The first was a cell-based assay in which most UGT2B7 was expected to remain as an oligomer. The substrate 4-methylumbelliferone (4-MU) was added directly to the medium of COS-1 cells expressing UGT2B7 and its mutants, and its enzyme activity was examined. Interestingly, although conversion to the metabolite 4-MU-β-D-glucuronide (4-MUG) was observed in UGT2B7 WT, the substitution of even one of the three cysteine residues with alanine reduced the catalytic ability of the mutant to below the detection limit (Fig. 9*A*). Western blot analysis confirmed that the number of UGT2B7 mutants expressed in the cells was not significantly different from that in the WT. This result suggests that the oligomer must be stable by disulfide bond(s) to exhibit enzyme activity; however, the possibility that the three cysteines may be important for the catalytic ability of UGT itself cannot be ruled out. Therefore, we next conducted *in vitro* assays using cell homogenates and mixed the enzyme source with 4-MU and UDPGA. Unlike cell-based experiments, UGT2B7 and its mutants were expected to largely dissociate into monomers during the preparation of homogenates from the cells. The results suggested that, as in the cell-based experiments, enzyme activity was observed only in the WT and was lost when even one cysteine was replaced by alanine. There was little difference in their expression levels in homogenates (Fig. 9*B*). The results of the two UGT assays suggest that these cysteine residues are important not only for oligomer stabilization but also for the catalytic ability of UGT.

## Discussion

In the present study, we characterized homo-oligomers of UGT2B7 using condition-optimized SDS-PAGE and chemical cross-linking. We were able to detect SDS-stable oligomers using non-reducing and non-heating SDS-PAGE (Fig. 2). SDS-stable oligomers of UGT have also been reported in previous studies (12, 13, 14). In all cases, SDS was used in sample preparation and/or the electrophoresis process. This may be important for the thorough solubilization and separation of UGT polypeptides on the gel, as we did not detect any UGT bands with Blue-Native PAGE using Coomassie Blue instead of SDS nor by Clear-Native PAGE using a detergent milder than SDS. Oligomers of other types of glycosyltransferases have also been detected using non-reducing SDS-PAGE (24, 25, 26). In particular, Sasai *et al.* detected SDS-stable oligomers of *N*-acetyl-glucosaminyltransferase V expressed in COS-1 cells using non-reducing SDS-PAGE, as was used in this study (25). The results obtained from UGT make it interesting to further explore whether other *N*-acetyl-glucosaminyltransferases require formation of SDS-stable oligomers for biological activity.

The addition of reducing agents greatly decreased the levels of SDS-stable oligomers, suggesting that disulfide bonds between UGT2B7 subunits are involved in their formation (Fig. 2). Further, we showed that Cys127, Cys156, and Cys282 are important for the formation of the inter-subunit disulfide bonds in UGT2B7 (Figure 3, Figure 4, Figure 5, Figure 6, Figure 7). The ER is thought to provide a more oxidative environment than the cytoplasm because it promotes the formation of disulfide bonds for protein folding (27, 28, 29). Hence, it has been hypothesized that the function of UGT is regulated by the redox balance in the ER through the formation of disulfide bonds. In support of this hypothesis, Ikushiro *et al.* showed that the reducing reagent, dithiothreitol, increased the glucuronidation ability of UGT1A6 in rat liver microsomes (30). They also raised the possibility that Cys121 and Cys125 in rat UGT1A6 form intra-molecular disulfide bonds, and the substitution of either cysteine residue with serine abolished its glucuronidation ability. Interestingly, the latter cysteine is conserved in major rat UGT isoforms and UGT1A6 isoforms in several species. In the present study, SDS-stable UGT2B7 oligomers were detected in several cell types (Fig. 2*C*), and the catalytic ability of UGT2B7 was lost when only one of the three cysteine residues was replaced with alanine (Fig. 9), further supporting our hypothesis. However, further studies, such as measuring UGT activity using cells with different ER redox levels or using methods that alter ER redox levels within cells, are necessary to demonstrate this.

The formation of UGT oligomers in living cells has previously been analyzed by FRET (15), and cross-linking with 1,6-bis(maleimido)-hexane revealed that UGT dimers are present in rat liver microsomes (8); however, the ratio of UGT monomers to oligomers in intact living cells is unknown. Hence, we utilized the chemical cross-linkers DSS and DSP, both of which are membrane-permeable, to analyze UGT oligomers as they exist in cells. The use of these reagents revealed for the first time that the majority of expressed UGT2B7 exists as oligomers rather than as monomers in COS-1 cells (Fig. 8). Based on homology modeling using structural data for several glucosyltransferases, Lewis *et al.* predicted that aromatic π-π interactions within the 183 to 200 region of UGT2B7 drive the formation of dimers (31). Together with this aromatic interaction, other intermolecular forces may contribute to the oligomerization of UGT2B7 in cells as the main driving force, and the inter-subunit disulfide bonds characterized in this study may stabilize some of the formed oligomers. Furthermore, the question of whether it is the monomers or oligomers that exhibit UGT activity is controversial. In cell-based experiments where the majority of UGT2B7 was expected to form oligomers, cells expressing UGT2B7 WT conjugated 4-MU (Fig. 9*A*), suggesting that UGT2B7 oligomers are enzymatically active.

UGTs form homo- and hetero-oligomers between subunits of the same or different isoforms, respectively. Although this study focused only on homo-oligomer formation of UGT2B7, Fujiwara *et al.* detected hetero-dimers formed from combinations of UGT1A1, UGT1A4, and UGT1A6 using SDS-PAGE, confirming that PAGE analysis is effective in the analysis of UGT oligomers (14). Hence, although methods for analyzing UGT oligomers are now well established, the physiological role of oligomerization remains unclear. UGT oligomer formation is not mere aggregation but has functional consequences, including alterations in enzyme function, such as the modulation of enzyme activities (14, 20, 21, 22, 32, 33, 34) and the acquisition of new substrate specificities (35). We have reported that, in addition to oligomer formation within the UGT family, UGTs also form complexes with P450 that can alter each other’s enzyme function (36, 37, 38). Based on the results of this study, we suggest that newly synthesized UGT first forms oligomers (Figs. 2 and 8) that can then interact with other oligomers to form higher-order complexes. The formation of oligomers presumably occurs during co-translation, as the mixing of microsomes expressing UGT2B21 and UGT2B22 individually, with detergent treatment, cannot achieve the substrate specificity acquired by co-expressing UGT2B21 and UGT2B22 (34). However, the possibility that UGT homo-oligomers separate into monomers and reform complexes with different partners to acquire the ability to metabolize a diverse range of chemicals remains inconclusive. Even though accumulating data suggest that hetero-oligomer formation of some UGT monomer combinations is important for acquiring new substrate specificities (32, 33, 34, 35), assays to determine UGT activity arising only from monomeric UGT have not been developed. The predominant status of UGT2B7 as an oligomer in living cells is evident from our current results when considering the chemical specificity of the cross-linkers used in this study (Fig. 8). Since protein disulfide isomerases that cleave disulfide bonds are located in the ER (39), the formation of inter-subunit disulfide bonds seems not to prevent such new pairings of UGTs. Backes’s group reported that a microdomain in the ER membrane can alter P450 function by affecting its protein-protein interactions (40, 41, 42). In the case of UGT, lipid-protein interactions may also trigger such dissociation and subsequent reassociation.

To the best of our knowledge, no method has been established to purify catalytically active recombinant UGT without an expression tag. Furthermore, the only knowledge we have of the structure of UGT has been derived from crystals of the C-terminal domain of UGT2B7 alone, without its transmembrane helix and cytoplasmic tail (5) and from NMR data (4). Therefore, extensive structure-based knowledge of UGT is still needed. Our findings in this study that inter-subunit disulfide bonds contribute to the stabilization of UGT oligomers and that cross-linkers can fix the natively formed inter-subunit UGT oligomers in cells should help with structure-based studies of UGT in the future.

## Experimental procedures

### Materials

Synthetic oligonucleotides were purchased from Fasmac (Kanagawa, Japan), and restriction enzymes were from Takara Bio (Shiga, Japan) or NIPPON GENE (Tokyo, Japan). The cross-linkers, DSS and DSP, were purchased from Thermo Fisher Scientific (Waltham, MA, USA). For the UGT assay, 4-MU, 4-MUG, and UDPGA trisodium salt were purchased from Nacalai Tesque (Kyoto, Japan) as the substrate, metabolite, and cofactor, respectively. All other reagents were of the highest quality commercially available.

### Construction of expression plasmids of UGT2B7 and its mutants

The open reading frames of UGT2B7 and UGT2B7-HA-tag were amplified by PCR using pFastBac1-UGT2B7-HA (23) as a template, KOD One PCR Master Mix (TOYOBO, Osaka, Japan) as the DNA polymerase, and the following primers: common forward, 5′-CGGGCCCCCCCTCGAAGGATGTCTGTGAAATGGAC-3′; UGT2B7 reverse, 5′-TCTAGAGTCGCGGCCCTAATCATTTTTTCCCTTCTTT-3′; UGT2B7-HA reverse, 5′-TCTAGAGTCGCGGCCTCAAGCGTAATCTGGAACAT-3′. The PCR product was cleaned up using the FastGene Gel/PCR Extraction Kit (NIPPON Genetics, Tokyo, Japan) and subcloned into the pEBMulti-Hyg vector (pEBM; FUJIFILM Wako Pure Chemical, Osaka, Japan) linearized with NotI/XhoI using the In-Fusion HD Cloning Kit (Takara Bio). Deletion and/or alanine-substituted mutants were generated using QuickChange site-directed mutagenesis. N1 (Δ253–529)/C1 (Δ24–200, TM) deletion constructs were amplified using pEBM-UGT2B7-HA as the template using the following primers: N1 forward, 5′-CCACTACATTATCTGAGACAATGGGGAAATACCCATACGATGTTC-3′; N1 reverse, 5′-GAACATCGTATGGGTATTTCCCCATTGTCTCAGATAATGTAGTGG-3′; C1 first round forward, 5′-GTTCCAGTACCACTCTTTGGATTACCCATACGATGTTC-3′; C1 first round reverse, 5′-GAACATCGTATGGGTAATCCAAAGAGTGGTACTGGAAC-3′; C1 second round forward, 5′-CTTTTGCTTTAGCTCTGGGAATTGTTTAACTGATCAAATGACTTTTATGG-3′; and C1 second round reverse, 5′-CCATAAAAGTCATTTGATCAGTTAAACAATTCCCAGAGCTAAAGCAAAAG-3′. N1 C127A and N1 C156A mutations were introduced using pEBM-N1 as the template and the following primers: C127A forward, 5′-GTCAATATTTGGTGACATAACTAGAAAGTTCGCTAAAGATGTAGTTTCAAATAAGAAATTTATG-3′; C127A reverse, 5′-CATAAATTTCTTATTTGAAACTACATCTTTAGCGAACTTTCTAGTTATGTCACCAAATATTGAC-3′; C156A forward, 5′-GCAGATGCTATTTTTCCCGCTAGTGAGCTGCTGGCTGA-3′; and C156A reverse, 5′-TCAGCCAGCAGCTCACTAGCGGGAAAAATAGCATCTGC-3′. The double mutant N1 C127/156A was also produced by sequentially introducing each mutation. The C282A mutation was introduced into each of the WT and C1 mutant proteins using the following primers: C282A forward, 5′-TGTTGGAGGACTCCACGCCAAACCTGCCAAACCC-3′ and C282A reverse, 5′-GGGTTTGGCAGGTTTGGCGTGGAGTCCTCCAACA-3′. Because the addition of the HA-tag to the C-terminus can affect enzyme activity (19), a series of mutants whose enzyme activities were measured (C127A, C156A, C282A, C127/156A, C127/282A, C156/282A, and C127/156/282A) were prepared using pEBM-UGT2B7 as a template and sequential alanine replacements with the primer pairs described above. The template plasmid was digested using DpnI treatment after amplification, and the mutation-introduced constructs were purified using the FastGene Gel/PCR Extraction Kit and transfected into ECOS Competent *E. coli* DH5α cells (NIPPON GENE). The other deletion mutants, C2–C6, were prepared as described previously (23). The nucleotide sequences of all constructs were verified by Sanger sequencing (Azenta Life Sciences Japan, Tokyo, Japan) and the NucleoBond Xtra Midi kit (Takara Bio) was used to prepare transfection-grade plasmid DNA.

### Cell culture, transfection, and lysate preparation

COS cells have long been used in UGT research (43), and our previous studies have shown that COS-1 cells express enough amounts of UGT to study enzyme activity and subcellular localization (19, 23), so we also used this cell line in this study. COS-1 cells were purchased from the Japanese Collection of Research Bioresources Cell Bank (Osaka, Japan) and grown in Dulbecco’s modified Eagle’s medium (DMEM; FUJIFILM Wako Pure Chemical, Cat#043–30085) supplemented with 10% fetal bovine serum (Corning Incorporated, Corning, NY, USA; Cat#35–079-CV). Cells were seeded in a 35 mm dish or 6-well plate 24 h before transfection. Polyethylenimine HCl MAX (PEI) was purchased from Polysciences (Warrington, PA, USA), dissolved into ultrapure water at 1 mg/ml, and sterilized using a syringe filter. The PEI solution (8 μl) and plasmid DNA (2 μg) were separately diluted with 150 μl of Opti-MEM I Reduced Serum Media (Opti-MEM; Thermo Fisher Scientific) and incubated for 5 min. The diluted PEI and plasmid DNA were gently mixed and further incubated for 10 min, after which the transfection reagent was added to the medium. Forty-eight hours after transfection, the culture medium was removed, and the cells were washed three times with phosphate-buffered saline (PBS). Then, 200 μl of RIPA buffer containing 50 mM Tris-HCl (pH 8.0), 150 mM sodium chloride, 0.5% sodium deoxycholate, 0.1% SDS, and 1.0% NP-40 was added and the cells were incubated for 15 min at 4 °C. The lysed cells were transferred to microtubes and centrifuged at 20,000 × *g* for 5 min at 4 °C. The resulting supernatants were collected as the cell lysates. The protein concentration was determined using Protein Assay Bradford Reagent (FUJIFILM Wako Pure Chemical, Cat#168–25911) with bovine serum albumin as a standard. HEK293 and HepG2 cells were also purchased from the Japanese Collection of Research Bioresources Cell Bank and handled under the same conditions as the COS-1 cells.

### Intercellular cross-linking

DSS and DSP were dissolved in DMSO, and a 20 mM solution was prepared. After 48 h of transfection, cells were washed twice with PBS and treated with PBS containing 1 mM DSS/DSP for 30 min. Tris-HCl (pH 7.5) was added to a final concentration at 10 mM to digest unreacted cross-linkers, and the cells were incubated for an additional 15 min. After a PBS wash, cell lysates were prepared as described above.

### SDS-PAGE and western blotting

Cell lysates (50 μg) were mixed with the same volume of 2 × SDS-PAGE sampling buffer containing 125 mM Tris-HCl (pH 6.8), 4% SDS, 20% glycerol, 10% 2-mercaptoethanol, and 0.01% bromophenol blue and heated at 90 °C for 3 min for the reducing and heating condition. For the non-reducing and non-heating conditions, 2-mercaptoethanol was replaced with purified water in the sample buffer and heating for 3 min was omitted. Samples were separated by SDS-PAGE on 7.5% acrylamide gels prepared with WIDE RANGE Gel Preparation Buffer (4×) for PAGE (Nacalai Tesque, Cat#07831–94), and electrophoresis was performed at room temperature for 70 to 150 min at a constant current of 20 mA per gel. Separated proteins were electroblotted onto a ClearTrans PVDF membrane (hydrophobic, 0.45 μM; FUJIFILM Wako Pure Chemical, Cat#034–25663), and the blot was washed with Tris-buffered saline containing 0.1% Tween 20 (TBS-T) and incubated in PVDF Blocking Reagent for Can Get Signal (TOYOBO, Cat#NYPBR01) for 30 min. After the blocking step, the blot was washed with TBS-T for 15 min and further incubated with the diluted primary antibody at 4 °C overnight. The primary antibodies utilized were: rabbit polyclonal anti-HA-tag antibody (Proteintech, Rosemont, IL, USA; Cat#51064-2-AP; RRID: AB_11042321), rabbit polyclonal anti-UGT2B7 antibody (Proteintech, Cat#16661-1-AP, RRID: AB_2214249), rabbit polyclonal anti-CNX antibody (Proteintech, Cat#10427-2-AP, RRID: AB_2069033), and rabbit polyclonal anti-GAPDH antibody (Proteintech, Cat#10494-1-AP, RRID: AB_2263076). The primary antibodies were diluted 5000 to 20,000-fold with Can Get Signal Immunoreaction Enhancer Solution (TOYOBO, Cat#NKB-101). After an overnight incubation, the blots were washed with TBS-T for 15 min and incubated for 1 h with horseradish peroxidase (HRP)-conjugated goat anti-rabbit secondary antibody (Jackson ImmunoResearch Labs, West Grove, PA, USA; Cat#111–035–003, RRID: AB_2313567) diluted 20,000-fold with Can Get Signal Immunoreaction Enhancer Solution. After a 15 min wash with TBS-T, the signal was visualized and quantified with EzWestLumi plus (ATTO, Tokyo, Japan) and an iBright Imaging System (Thermo Fisher Scientific) as the substrate of HRP and detector, respectively. The used blots were incubated with Stripping Solution (FUJIFILM Wako Pure Chemical, Cat#193–16375) at room temperature for 10 min to detect another target on the same membrane. The blots were then washed with PBS for 15 min and incubated with the primary antibody for another target.

### UGT assay

The enzymatic activity of UGT2B7 was measured using two approaches: a cell-based assay, in which intracellular oligomers are expected to be maintained, and an *in vitro* assay using cell homogenates, in which the majority of UGT is present in the monomeric form. For the cell-based assay, COS-1 cells (1.0 × 10^5^ cells/well) were seeded into 24-well plates 24 h before transfection. The PEI solution (4 μl) and plasmid DNA (1 μg) were incubated in 50 μl Opti-MEM, and the mixture was added to the culture medium. The cells were maintained for 48 h, and then the medium was changed to DMEM containing 500 μM 4-MU. After 24 h, a part of the culture medium (300 μl) was collected, mixed with 150 μl of methanol, and centrifuged at 20,000 × *g* for 20 min at 4 °C. The supernatant was collected for subsequent analysis with high-pressure liquid chromatography (HPLC). The enzyme activity was determined for each well.

The homogenate was used as the enzyme source in the *in vitro* assays. The transfected cells under the same conditions as for lysate preparation were suspended in PBS containing 10% glycerol, homogenized using an ultrasonic homogenizer VP-050 (TAITEC, Saitama, Japan), and centrifuged at 500 × *g* for 20 min at 4 °C. The supernatant was collected as homogenates. Homogenates were treated with alamethicin (50 μg alamethicin/mg homogenates) for 20 min on ice to allow UGT2B7, located on the luminal side of the ER, to easily access the substrates and UDPGA. The reaction mixture (total volume: 300 μl) contained 100 mM Tris-HCl (pH 7.5), 10 mM MgCl_2_, 300 μM 4-MU, 300 μg of alamethicin-treated homogenates, and 2 mM UDPGA. The reaction was started by adding UDPGA after 10 min preincubation at 37 °C. After incubation at 37 °C for 60 min, the reaction was terminated by adding 150 μl of methanol, and the sample was centrifuged at 20,000 × *g* for 20 min at 4 °C to remove protein. The supernatants were collected for HPLC. Enzyme activity was determined using relative UGT, a western blot-based quantitative value, where the expression levels of UGT2B7 and its mutants were first corrected for the level of GAPDH, and then the relative value of UGT2B7 WT was defined as 1.0. However, since the production of 4-MUG was below the detection limit for all the mutants, it was not detectable (ND) except for in the WT.

### HPLC analysis

The HPLC system consisted of a Shimadzu Prominence series (Shimadzu, Kyoto, Japan) with an LC-20AD pump, CTO-20A column oven, and SPD-20A UV-VIS detector. The HPLC sample (100 μl) was injected and separated with TSKgel ODS-80Ts column (4.6 mm × 15 cm, 5 μM; Tosoh, Tokyo, Japan), and the mobile phase consisted of 70% 10 mM sodium phosphate buffer (pH 2.6) and 30% acetonitrile at a constant flow rate of 0.5 ml/min. Absorbance was measured at 320 nm. The retention time of 4-MUG under these conditions was 5.1 min, and quantification was based on a standard curve prepared using the 4-MUG standard.

### Statistical analysis

Analyses were performed using GraphPad Prism 5.04 software (GraphPad Software, La Jolla, CA, USA). The effect of cellular crosslinking with DSS or DSP on UGT2B7 oligomers was estimated by quantifying the UGT2B7-HA monomer. The protein levels of the UGT2B7-HA monomers were first determined as % GAPDH, and the monomer levels in the DSS- and DSP-treated groups were calculated with and without reduction, respectively, based on the monomer level in the DMSO-treated group as 100%. The experiment was performed independently three times, and significant changes in monomer levels were determined using one-way analysis of variance, followed by Tukey’s test (∗*p* < 0.05; ∗∗*p* < 0.01; ∗∗∗*p* < 0.001).

## Data availability

All data are presented in this manuscript.

## Supporting information

This article contains supporting information.

## Conflict of interest

The authors declare that they do not have any conflicts of interest with the content of this article.
