# Supplementary material for: Disulfide bond-mediated stabilization of the oligomers of UDP-glucuronosyltransferase 2B7
Source: J Biol Chem. 2025 Jul 21;301(9):110502. doi: 10.1016/j.jbc.2025.110502 (PMC12390933; doi:10.1016/j.jbc.2025.110502)
Supplement: Supporting information [file mmc1.pptx]

## Slide 1
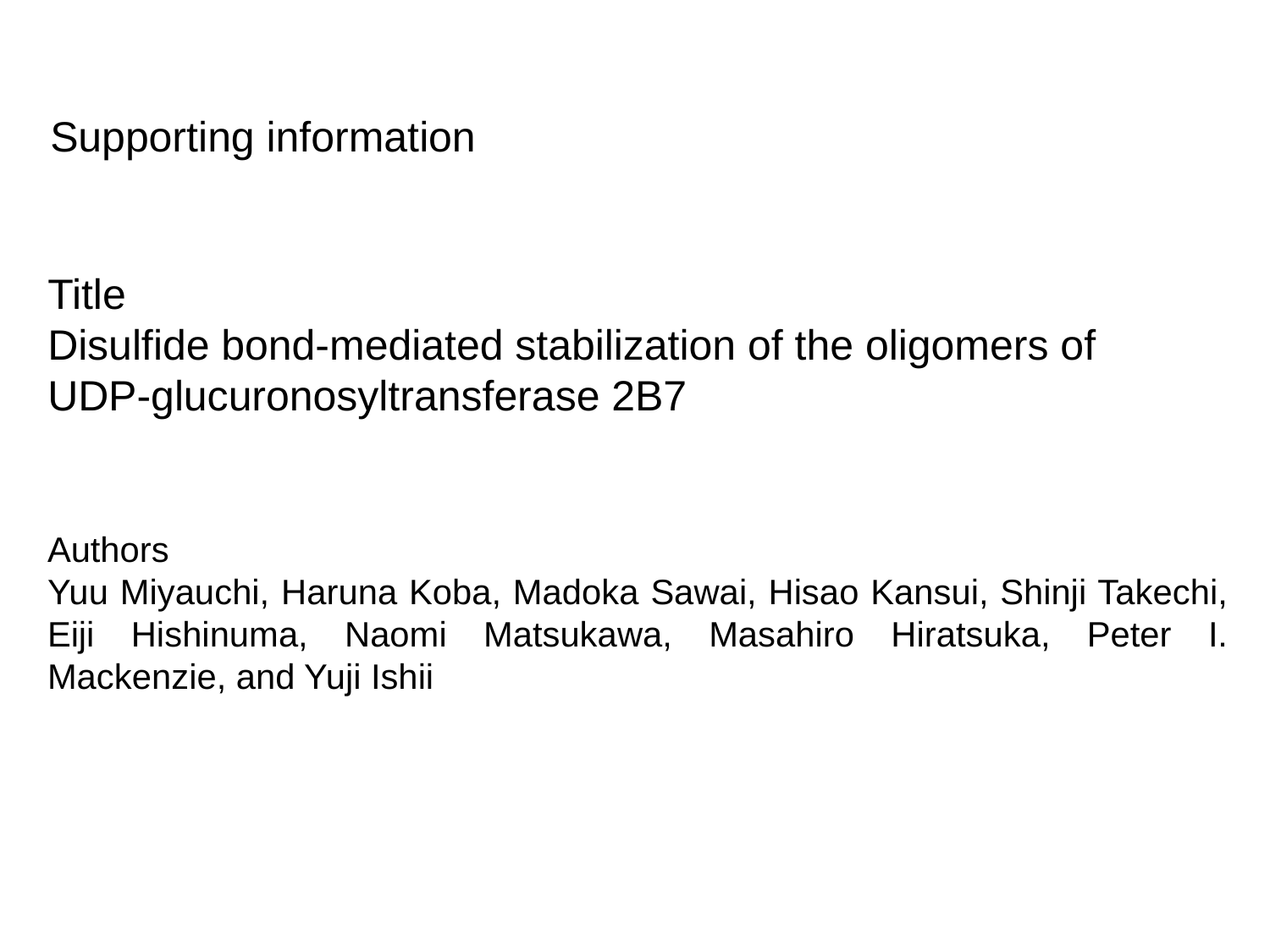

Supporting information
Title
Disulfide bond-mediated stabilization of the oligomers of UDP-glucuronosyltransferase 2B7
Authors
Yuu Miyauchi, Haruna Koba, Madoka Sawai, Hisao Kansui, Shinji Takechi, Eiji Hishinuma, Naomi Matsukawa, Masahiro Hiratsuka, Peter I. Mackenzie, and Yuji Ishii

## Slide 2
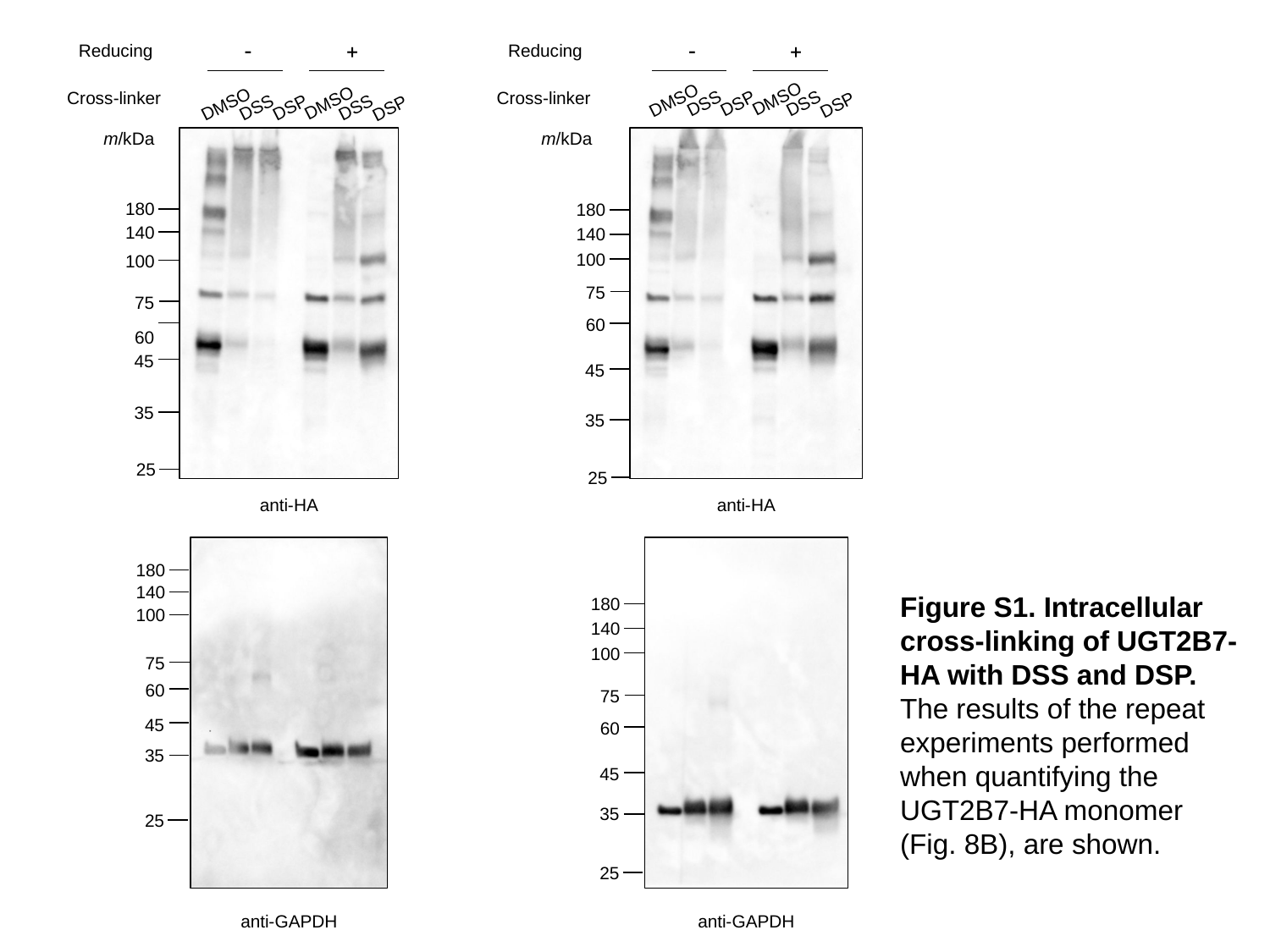

−
+
−
+
Reducing
Reducing
Cross-linker
Cross-linker
DMSO
DMSO
DMSO
DSS
DSP
DSS
DMSO
DSP
DSS
DSP
DSS
DSP
m/kDa
m/kDa
180
180
140
140
100
100
75
75
60
60
45
45
35
35
25
25
anti-HA
anti-HA
180
140
Figure S1. Intracellular cross-linking of UGT2B7-HA with DSS and DSP.
The results of the repeat experiments performed when quantifying the UGT2B7-HA monomer (Fig. 8B), are shown.
180
100
140
100
75
60
75
45
60
35
45
35
25
25
anti-GAPDH
anti-GAPDH
